# Superior Mechanical Properties of Double-Network Hydrogels Reinforced by Carbon Nanotubes without Organic Modification

**DOI:** 10.3390/ijms141122380

**Published:** 2013-11-13

**Authors:** Weifu Dong, Chiguang Huang, Yang Wang, Yujie Sun, Piming Ma, Mingqing Chen

**Affiliations:** The Key Laboratory of Food Colloids and Biotechnology, Ministry of Education, School of Chemical and Material Engineering, Jiangnan University, Wuxi 214122, China; E-Mails: s110608008@vip.jiangnan.edu.cn (C.H.); justwangyang@126.com (Y.W.); ytsunyujie@126.com (Y.S.); p.ma@jiangnan.edu.cn (P.M.)

**Keywords:** nanocomposite hydrogels, double network, CNTs, mechanical strength

## Abstract

A facile method is developed to fabricate nanocomposite double-network (DN) gels with excellent mechanical properties, which do not fracture upon loading up to 78 MPa and a strain above 0.98, by compositing of carbon nanotubes (CNTs) without organic modification. Investigations of swelling behaviors, and compressive and tensile properties indicate that equilibrium swelling ratio, compressive modulus and stress, fracture stress, Young’s modulus, and yield stress are significantly improved in the presence of CNTs. Scanning electron microscopy (SEM) reveals that the pore size of nanocomposite DN gels is decreased and some embedded micro-network structures are observed on the fracture surface in comparison to DN gels without CNTs, which leads to the enhancement of mechanical properties. The compressive loading-unloading behaviors show that the area of hysteresis loop, dissipated energy, for the first compressive cycle, increases with addition of CNTs, which is much higher than that for the successive cycles. Furthermore, the energy dissipation mechanism, similar to the Mullins effect observed in filled rubbers, is demonstrated for better understanding the nanocomposite DN polymer gels with CNTs.

## 1. Introduction

Polymer hydrogels are soft, three-dimensional cross-linked macromolecular networks with large amount of water and have potential applications in many fields, such as cell-sheet cultivation [1], drug delivery [2–4], contact lenses [5], anti-fouling coatings [6], superabsorbants [7], *etc.* However, most polymeric hydrogels with high water content have inherent fragility, which limits their applications as load bearing materials [8], bioactuatior [9,10], biosensor [11], microfluid [12], and tissue engineering [13]. Recently, several designed hydrogels with high mechanical performance have been reported, such as slide-ring hydrogels [14], nanocomposite hydrogels [15], double network (DN) hydrogels [16], tetra-PEG hydrogels [17], macromolecular microsphere composite hydrogels [18], and hydrophobic association hydrogels [19]. Among them, there has been considerable interest in DN gels due to their unique network structure, interpenetrating polymer network (IPN), which is similar to articular cartilage. Therefore, the subtle combination of soft and hard networks designed in DN gels has potential application in tissue substitutional materials. It is reported that the DN gels exhibit much higher strength and toughness than the individual component does, and the fracture stress and toughness of them are in the order similar to the soft bio-tissues [20].

Nanocomposite (NC) gels are new class of hydrogels and consisting of organic/inorganic network structure with outstanding mechanical properties [15,21–23]. Normally, the nanoparticles within NC gels are considered as cross-linkers by covalent bonding or physical adsorption with polymer chains. The resulting NC gels overcome most of the disadvantages associated with conventional cross-linked hydrogels, such as mechanical fragility, structural heterogeneity, and slow de-swelling rate. Gaharwar *et al.* have developed a range of hydroxyapatite nanoparticles (nHAp) filled poly(ethylene oxide) (PEO) to form highly elastic nanocomposites hydrogels, and investigated the cross-linking effect of nHAp on the structure and mechanical properties of the hydrogels [24]. More recently, Alba Marcellan *et al.* have coupled a poly(*N*,*N*-dimethylacrylamide) covalent network with silica nanoparticles as physical cross-linkers and proposed a dissipative mechanism for nano-hybrid hydrogels [25]. The significantly enhanced mechanical properties of the hybrid hydrogels are attributed to a unique dissipation by the strong reversible adsorption/desorption interactions between nano-silica particles and network chains during deformation and fracture processes.

As mentioned above, it is observed that DN and NC gels exhibit the enhanced mechanical properties. Nevertheless, there is yet a challenge to further improve the mechanical properties of hydrogels in order to satisfy the high demand in some fields such as bio-tissue. Fu *et al.* have combined the advantages of DN gels and NC gels to achieve silica-grafted DN gels with high mechanical properties [26], in which the functional silica nanoparticles are used as macro-crosslinkers to copolymerize with the first network and subsequently swollen to host the polymerization of a second network. Recently, many researches are focused on silica, clay, and graphene oxide (GO) nanocomposite DN gels [27–31], however little concern is involved in CNTs reinforced DN gels. In this work, based on the nanocomposite double-network hydrogels principle, we presented a facile method to fabricate CNTs-filled DN gels with excellent mechanical strength, which is comparable with the aforementioned silica-grafted DN gels. Noteworthy, CNTs without organic modification are very effective to reinforce DN gels, even there is not covalent bonding, but physical absorption between CNTs and polymer DN gel chains.

In our paper, an individual poly(2-acrylamido-2-methylpropanesulfonic acid) (PAMPS) hydrogels by compositing CNTs was fabricated as the first nanocomposite network and swollen to balance in acrylamide solution, then polymerized to form the second network. Finally, PAMPS/PAAm/CNTs nanocomposite DN hydrogels was prepared. Furthermore, the influences of the CNTs on the mechanical properties, microstructure, and loading-unloading compression behaviors of nanocomposite DN hydrogels were investigated.

## 2. Results and Discussion

### 2.1. Swelling Behavior

**Figure 1:**
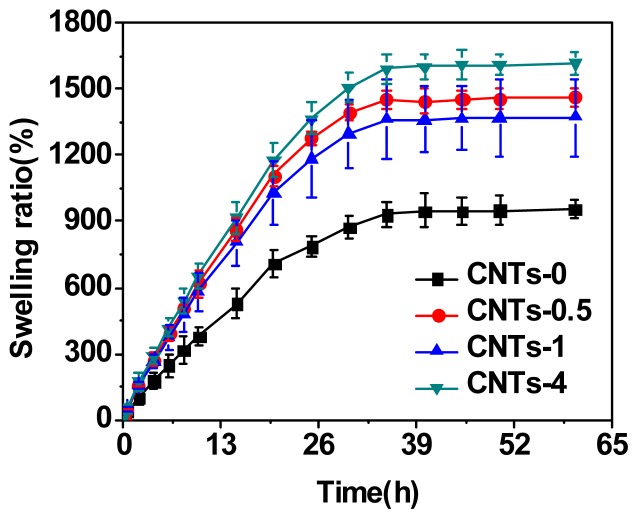
The swelling curves of nanocomposite DN hydrogels composited with various CNTs contents.

The swelling behaviors of the CNTs-filled DN hydrogels with different CNTs content were shown in Figure 1. The swelling ratios of the hydrogels in water increased with time going until reaching equilibrium at about 35 h. The equilibrium-swelling ratio (ESR) of the CNTs-filled DN gels was much higher than that of DN gels, and further enhanced with increase to the content of CNTs. With the CNTs content of 4 wt%, the ESR reached 1600%, which was much higher than the DN gel without CNTs. The ESR of nanocomposite DN gels with 0.5 and 1 wt% are similar and lower than that of DN gels with 4 wt%. CNTs are easily to form physical cross-linkers and enhance cross-linking degree of DN network. Normally, the ESR decreases with increase of cross-linking degree. Covalent or physical cross-linking restricts the configuration of the swollen nanocomposite hydrogels in aqueous media. However, for DN gels of this work, the ESR improved with increase of cross-linking degree. It was inferred that CNTs could occupy the void space in PAMPS gels, which provided the space to contain more water during swelling and resulted in a more porous and higher swelling degree network. This phenomenon was also observed in nanocomposite DN gels with silica-particles elsewhere [26,32].

### 2.2. Mechanical Properties

**Figure 2:**
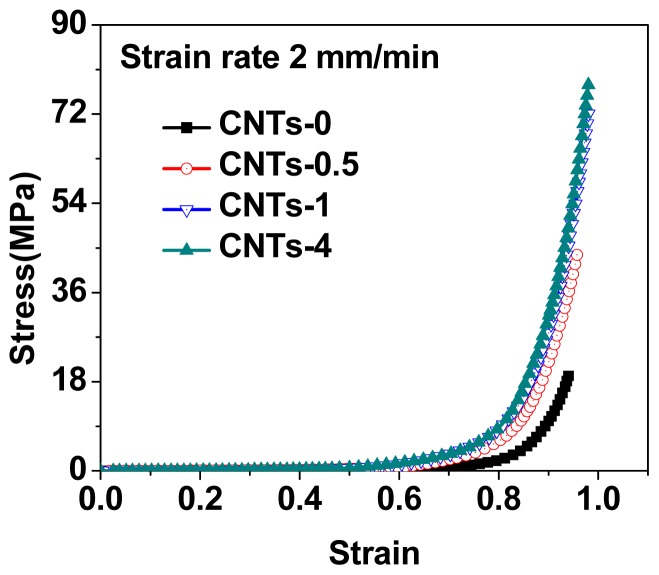
Compressive stress-strain curves for nanocomposite DN hydrogels.

**Figure 3:**
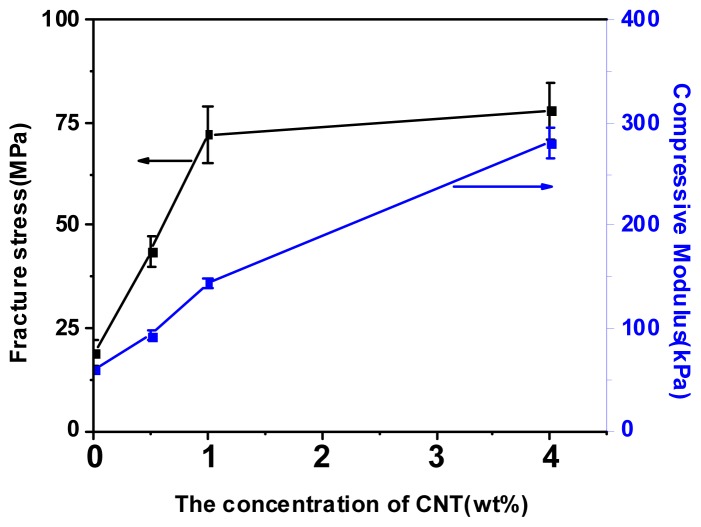
The fracture stress and compressive modulus of nanocomposite DN gels as a function of CNTs content.

The compressive stress-strain curves were shown in Figure 2, and the data of mechanical properties were summarized in Table 1. It was shown that nanocomposite DN gels with CNTs possessed excellent compressive properties. The compressive modulus of CNTs-filled DN gels (CNTs, 4 wt%) was 280 ± 14 kPa, leading to a nearly four-fold increase, compared with neat DN gels (60 ± 2 kPa). Moreover, when the content of CNTs was 1 wt%, the fracture stress of CNTs-filled DN gels significantly increased from 19 to 72 MPa at a strain of 0.98. Obviously, the compressive modulus and fracture stress of nanocomposites with 1 wt% and 4 wt% were significantly higher when compared with the control (*p* < 0.05, ANOVA). As shown in Figure 3, with further increase to the amount of CNTs, the fracture stress did not enhance sharply and level off, but the compressive modulus kept a constant increase, up to the content of 4 wt%. On the contrary, the mechanical properties for silica-grafted DN gels decreased with further increasing the amount of silica nanoparticles [26]. It was clear that the PAMPS/PAAm/CNTs nanocomposite DN hydrogels exhibited superior strength and toughness, and CNTs were very effective to reinforce the DN gels.

**Figure 4:**
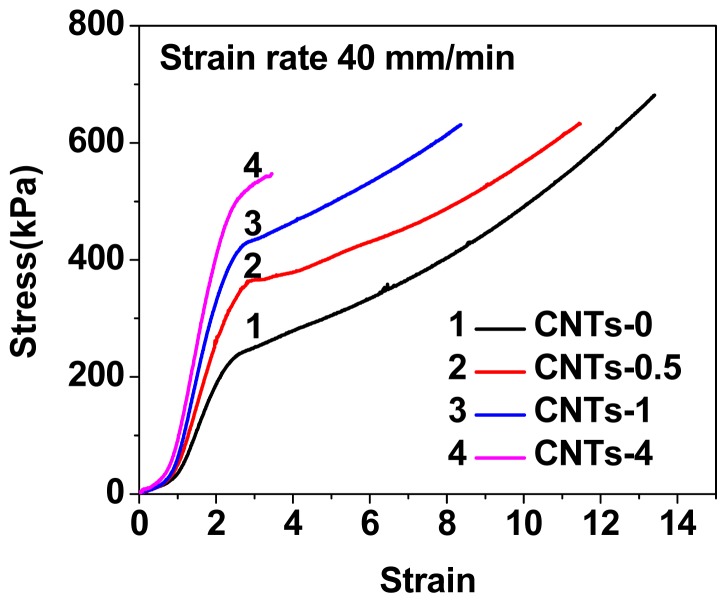
Tensile stress-strain curves of DN and nanocomposite DN hydrogels.

The tensile stress-strain curves were shown in Figure 4, and the data of Young’s modulus, yield stress, ultimate stress, and ultimate strain for the neat and nanocomposites DN gels were listed in Table 2. Clearly, the addition of CNTs led to an increase in Young’s modulus and yield strength, but a decrease in ultimate strain. The Young’s modulus for all samples were different from each other (*p <* 0.05, ANOVA). The Young’s modulus increased from 20.8 kPa for neat DN gel to 35.6 kPa for CNTs-4. The increase in stiffness might be explained as the following two, important, points: (1) There are the strong physical interactions between polymer chains (PAMPS and PAAm) and CNTs, and CNTs can be used as physical cross-linkers; and (2) CNTs are easily entangled themselves to form physical cross-linkers, and reinforce the DN gels.

### 2.3. Morphology

**Figure 5:**
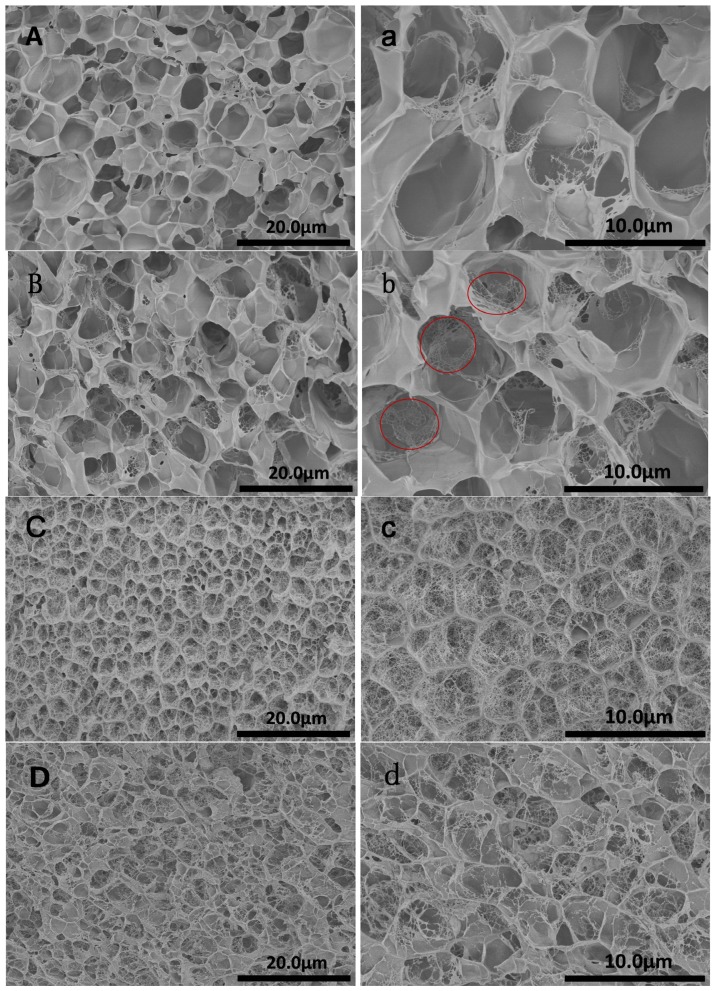
SEM images of nanocomposite DN hydrogels (**A**,**a**) CNTs-0; (**B**,**b**) CNTs-0.5; (**C**,**c**) CNTs-1 and (**D**,**d**) CNTs-4. The embedded micro-network structures were shown in the red circles.

The porous microstructures of nanocomposite double-network hydrogels with different CNTs contents were shown in Figure 5. It was seen that CNTs could dramatically decrease the size of pore. When the content of CNTs was 1 wt%, the pore sizes decreased to 5 ± 1 μm, in comparison with 12 ± 2 μm for PAMPS-PAAM DN gels. With further increasing of the amount of CNTs, the pore size did not decrease more, and became slightly irregular. Normally, the smaller pore sizes indicated denser networks, which showed relatively higher mechanical strength [33]. For example, the compressive stress of PAMPS-PAAM DN gels was 19 MPa at a fracture strain of 0.94, whereas that of nanocomposite DN hydrogel with 4 wt% CNTs reached 78 MPa without breaking at a strain of 0.98.

**Figure 6:**
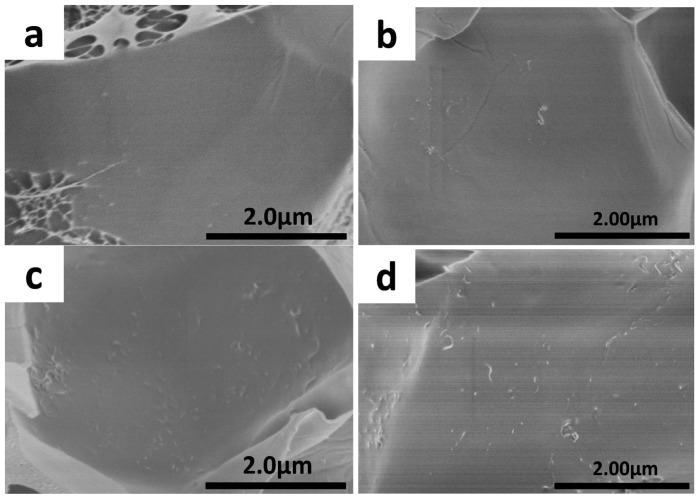
SEM images of pore wall for CNTs-filled nanocomposite hydrogels (**a**) CNTs-0; (**b**) CNTs-0.5; (**c**) CNTs-1; and (**d**) CNTs-4.

Moreover, with the addition of CNTs, the fracture surface became rough and embedded micro-network structures (within the red circles of Figure 5b) were formed. It was interesting that, when the content of CNTs was 1 wt%, there were a large amount of micro-network structures located in the pores and the thickness of walls got thicker. When the concentration of CNTs was increased to 4 wt%, the amount of micro-network structures became less. The similarly detail reasons were described in the research work of Fu *et al.* [26]. As we know, the porous structures of double-network hydrogels are generated due to phase separation between the crosslinks and water. CNTs can act as crosslinkers in DN gels that immobilize part of the networks during swelling. That is, additional phase separation may occur between normal crosslinks and the immobile CNTs, which may lead to the formation of micro-network structures at appropriate CNT contents. With high CNT contents, both the mobile and immobile crosslinks may match to yield a rigid network with high elastic energy so that the phase separation becomes less. Thus, the amount of micro-network structures CNTs-4 became less. As reported previously, the formation of embedded micro-network structures led to increase in the compressive strength and toughness [26,34–36]. However, the compressive strength of CNTs-4 was higher than that of CNTs-1, which was attributed to the stronger reinforced effect of CNTs at higher concentration. The high magnification of the pore walls was shown in Figure 6. It was evident that CNTs were embedded in the pore walls of nanocomposite DN gel and fairly well dispersed.

### 2.4. Compressive Loading-Unloading Behaviors and Analysis

**Figure 7:**
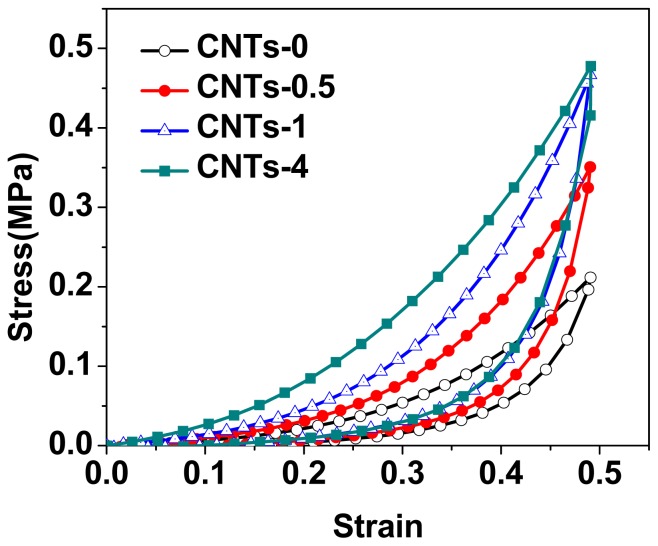
Effect of CNTs contents on the first compressive loading-unloading cycles.

**Figure 8:**
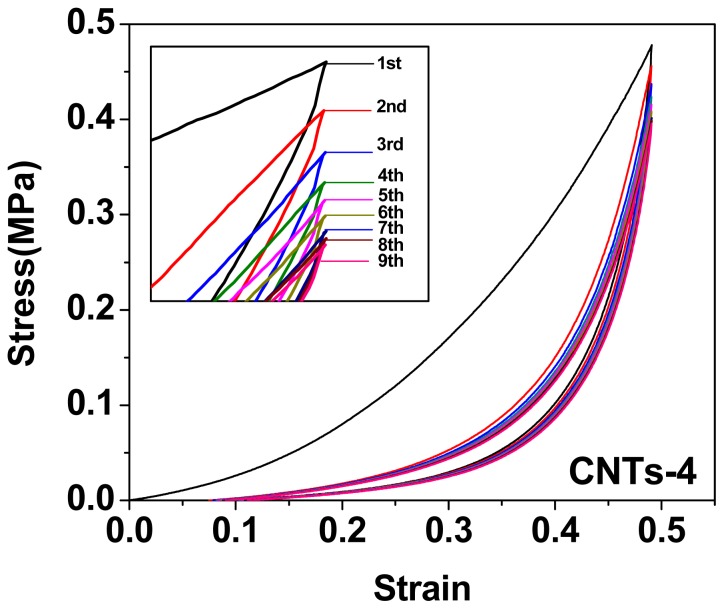
General shape of nine consecutive compressive loading-unloading cycles is given for CNTs-4.

Strain-controlled compressive loading-unloading curves were plotted in Figures 7 and 8. Compressive strain from 0 to 0.5 was applied, since this range was involved in the particle-polymer interaction but not the possible particle-particle compression. As shown in Figure 7, the loading/unloading curves of CNTs-filled DN gels indicated typical hysteresis. In all cases, the loading curve of the first compressive cycle was different from the unloading curve, and so was the successive loading curves. On the first compressive loading-unloading cycle, the area of hysteresis loop, dissipated energy, for all samples, were significantly different from each other (*p <* 0.05, ANOVA) and enhanced with increasing the amount of CNTs. When the concentration of CNTs was 4 wt%, the dissipated energy of nanocomposite DN gels was increased to 48.4 ± 1.5 kJ/m^−3^, in comparison with 13.1 ± 0.8 kJ/m^−3^ for DN gels, which was due to enhanced cross-linking degree of the first PAMPS network by addition of CNTs. Compared with the first loading-unloading cycle, the hysteresis loss of the second compressive cycle decreased sharply (Table 3 and Figure 8). However, the successive one diminished slowly, implying there was a slow dissipation during the subsequent compressive cycles. That was to say, these successive compressive cycles seemed to nearly overlap in the compressive stress-strain curves, exhibiting better elasticity, which were very similar to the filled rubbers [37]. Moreover, the dissipated energies of the successive loading-unloading cycles for nanocomposite DN gel were larger than that of neat PAMPS/PAAm DN gel, which also proved that PAAm macromolecule chains were entangled with CNTs and formed some physical crosslinkers.

The irrecoverable and large hysteresis loss of first compressive cycle, rather different from the rubber-like behavior of the subsequent cycles, resulted in sacrificing the integrity of the first PAMPS network. In order to investigate the enhancement of dissipated energy for primary network with CNTs, quantitative analysis for the hysteresis loss was carried out. In this study, viscoelastic hysteresis is likely to be negligible due to the low viscosity of DN gels and assumed that dissipated energy is caused by bond fracture. The dissipated energy of classical DN gels is quite in line with the Lake-Thomas elastic model prediction. Therefore, the Lake-Thomas model is applied for estimating Φ_f_ that is the fraction of covalent bonds in the primary network that have become unstressed, either broken or no longer load bearing, during the deformation process [37]. Φ_f_ is calculated according to the following equation:

(1) $$\Phi_{f}=\frac{E_{d}}{C_{c-c}\times E_{\text{f}}}\times 100\%$$

where *E*_d_ represent the hysteresis loss or dissipated energy calculated according to the area of hysteresis loop on first compressive loading-unloading cycle. *C*_c-c_ (120 mol/m^3^) is the molar concentration of carbon-carbon covalent bond in the primary network, and *E*_f_ (360 kJ/mol) is the fracture energy to a carbon-carbon bond [37]. It is noted that the highly cross-linked PAMPS network is broken first, followed by the extension of loose PAAm network in the DN gels. Φ_f_ is described the energy release of bond breakage during the deformation process. In other words, it represents the number of available broken chains between two cross-linkers in PAMPS network. During the fracture process, these available broken chains in primary network are significant for load-bearing and energy dissipated of the DN gels.

**Figure 9:**
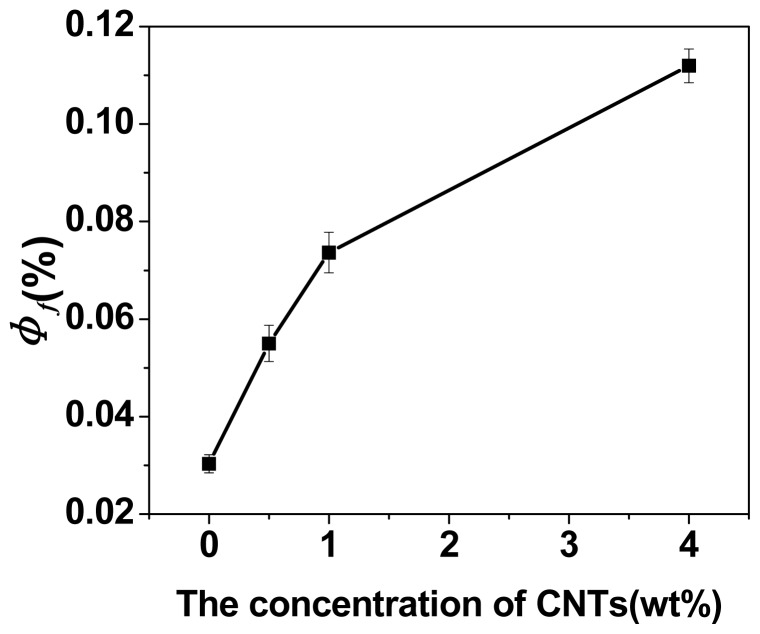
Fraction of the fractured bonds on a strain of 0.5 during the first compressive loading cycle as a function of the concentration of CNTs.

The values of Φ_f_ achieved from the first compressive loading-unloading cycle as a function of the concentration of CNTs were showed in Figure 9. The values of Φ_f_ for all samples were significantly different from each other (*p <* 0.05, ANOVA). It was observed that the Φ_f_ increased from 0.031 to 0.112 when the content of CNTs increased from 0 to 4 wt%. The increase of Φ_f_ demonstrated the dissipated energy of nanocomposite DN hydrogels was enhanced during the deformation process in the presence of CNTs, which was related to the larger amount of elastic chains based on the higher cross-linking density. In conclusion, an enhancement in Φ_f_, as well as compressive elastic modulus and compressive strength on first cycle, suggested that nanocomposite DN hydrogels with the CNTs-embedded PAMPS sheets as the first network were effective for the dissipation of energy and resistance of fracture, in comparison with conventional DN gels.

## 3. Experimental Section

### 3.1. Materials

2-Acrylamido-2-methylpropanesulfonic acid (AMPS, 98%), N,N′-methylene-bis-acrylamide (MBAA, 99.0%), acrylamide (AAm, A.R.), potassium persulfate (KPS, A.R.), and sodium dodecylbenzenesulfonate (SDBS, 95.0%) were purchased from Aladdin Chemistry Co. Ltd., Shanghai, China, and used as received. Multi-walled carbon nanotubes (CNTs) was supplied by Tsinghua University and used as received.

### 3.2. Experimental Section

The nanocomposite DN hydrogels were prepared by a two-step free-radical polymerization in aqueous medium. The nomenclature and compositions of nanocomposite DN hydrogels were summarized in Table 4. Firstly, an aqueous dispersion of CNTs was prepared by dispersing multi-walled carbon nanotubes into SDBS aqueous solution and sonicated for 1 h. The cross-linking agent *N*,*N*′-methylene-bis-acrylamide (MBAA), the initiator potassium persulfate (KPS), and the monomer 2-acrylamido-2-methylpropanesulfonic acid (AMPS) were added into an aqueous dispersion of CNTs (varied at 0.5, 1 and 4 wt% with respect to the weight of AMPS). Subsequently, the mixture was reacted at 60 °C for 10 h into a reaction container made up of two glass plates and a silicone rubber gasket to synthesize the PAMPS/CNTs hydrogel. Secondly, the nanocomposite PAMPS gel was put into an acrylamide (AAm) aqueous solution containing *N*,*N*′-methylene-bis-acrylamide (MBAA) and potassium persulfate (KPS), and immersed for one day. The swollen PAMPS gel was synthesized at 60 °C for 10 h in a reaction container to yield a PAMPS/PAAm/CNTs nanocomposite double-network hydrogel. For all these synthesis, the [AMPS]/[MBAA]/[KPS], [AAm]/[MBAA]/[KPS], and [AMPS]/[AAm] molar ratios were fixed at 1/0.04/0.005, 2/0.001/0.002, and 1/2, respectively.

### 3.3. Measurements and Characterization

#### 3.3.1. Scanning Electron Microscopy (SEM)

The micromorphology of hydrogel samples was evaluated using a Hitachi S4800 scanning electron microscope (Tokyo, Japan) at an accelerating voltage of 10 kV. Firstly, the hydrogel samples were cut into 20 × 2 mm^2^ pieces, and rapidly quenched into liquid nitrogen and freeze-dried. Then, the samples were fractured using a cold scalpel after freeze-dried. The fractured surfaces of cross-section were observed by SEM after sputter-coating with a thin gold layer.

#### 3.3.2. Swelling Experiments

The swelling behaviors of hydrogels were measured in triplicate by immersing them in a large excess of water at room temperature to reach swelling equilibrium. As-prepared five cylindrical samples with 20 mm length, 20 mm width, and 10 mm height were used for the swelling experiments. The swollen samples were weighed at set intervals followed by blotting off the excess water from the sample surface with a filter paper. The swelling ratios (SR) were calculated by the following equation, *SR =* (*M*_s_ − *M*_d_)/*M*_d_ × 100%, where *M*_s_ and *M*_d_ were the weights of the swollen and corresponding dried hydrogels, respectively.

#### 3.3.3. Tensile Measurements

Tensile tests were measured with an electronic universal material testing machine (KDIII, Kaiqiangli Co. Ltd., Shenzhen, China) at room temperature. The testing samples were cut into a dumbbell shape as sizes of 50 mm in length, 10 mm in width, 2~3 mm in thickness, 10 mm in gauge length, and 4 mm in inner width. For tensile tests, an elongation speed of 40 mm/min was applied to determine the tensile properties of each sample. Elastic modulus, yield stress, fracture stress, and ultimate strain were calculated from the stress-strain measurement. At least three specimens were tested for each hydrogel. The fracture stress, defined as engineering stress, was determined as the stress at breaking point. The ultimate strain was determined as the strain at breaking point. The initial slope at strain between ɛ = 1 and 2 of the first loading stress–strain curve was used to calculate the initial elastic modulus. The yield stress was determined as the point at which slope of stress–strain curves becomes zero, if such point was not observed, the yield stress was estimated as the point where the slope of the stress–strain curve changed dramatically [32].

#### 3.3.4. Compressive Measurements

Compressive tests were measured with an electronic universal material testing machine (KDIII, Kaiqiangli Co. Ltd., Shenzhen, China) at room temperature. The cylindrical sample of 8 mm in diameter and 4~6 mm in thickness was set on the lower plate and compressed by the upper plate, which was connected to a load cell, at a strain rate of 2 mm/min. Elastic modulus and compressive stress were calculated on basis of the compressive stress-strain testing. At least three specimens were tested for each hydrogel. The compressive stress, defined as engineering stress, was approximately calculated as σ_c_*= F*/π*R*^2^, where *R* is the original radius of the specimen. The strain under compression is defined as the change in the thickness *h* relative to the original thickness *h*_o_ of the freestanding specimen, ɛ = (*h*_o_ − *h*)/*h*_o_. The stress and strain between ɛ = 0 and 0.1 were used to calculate the initial elastic modulus [26].

Compressive loading–unloading cycles were performed in order to investigate the hysteresis (or dissipated energy) of the viscoelastic processes by using a compression speed of 2 mm/min. Compressive cycles were applied with the strain from 0 to 0.5. The compressive stress, defined as engineering stress, was determined as the point at a strain of 0.5 on compressive loading curve. The compressive modulus is calculated from the slope at low strain between ɛ = 0 and 0.1. The hysteresis loop or dissipated energy was determined as the area of compressive loading-unloading cycle.

#### 3.3.5. Statistical Analysis

Data are showed as mean ± standard error of the mean values. Statistical analysis was performed using Origin (version 8; OriginLab Corp, Northampton, MA, USA) to determine the statistical differences. Statistical comparisons were performed with one-way analysis of variance (ANOVA) for an average of three to six replicates. Statistical significance for all tests was set to be at a *p* value < 0.05.

## 4. Conclusions

The PAMPS/PAAm/CNTs nanocomposite DN gels had been successfully prepared by a two-steps solution radical polymerization in aqueous media. With the addition of CNTs, the equilibrium-swelling ratio of nanocomposite DN gels increased greatly. The measurement of mechanical properties indicated that compressive modulus and stress, fracture stress, Young’s modulus, and yield stress were dramatically improved in the presence of CNTs. The fracture stress and compressive modulus of nanocomposite DN gels reached 78 MPa at a strain of 0.98 and 280 kPa, respectively, leading to an increase of four-fold times compared with neat PAMPS/PAAm DN gels (19 MPa and 60 kPa, respectively). The results of microscopy displayed that the pore size became smaller and the embedded micro-network structures appeared on the fracture surface of nanocomposite DN gels by addition of CNTs, which accounted for an improvement of mechanical properties. The compressive loading–unloading behaviors showed the dissipated energy of the first compressive cycle was much higher than that of the successive cycles, and enhanced with further increasing of the amount of CNTs. The quantitative analysis of dissipated energy and fraction of broken covalent bonds gave a fully understanding in the influence of CNTs on the compressive properties. In conclusion, PAMPS/PAAm/CNTs nanocomposite DN gels have super strength and toughness, which provide us a new idea to prepare the DN gels with excellent mechanical properties.

## Figures and Tables

**Table 1:** The compressive properties of nanocomposite double-network (DN) hydrogels.

Samples	Compressive properties			

	Compressive modulus (kPa)	σ_c,0.9_ (MPa)	Fracture stress	Fracture strain
CNTs-0	60 ± 2	10.09 ± 1.1	19.17 ± 3.1	0.94 ± 0.01
CNTs-0.5	93 ± 5	22.02 ± 2.9	43.61 ± 3.9	0.95 ± 0.01
CNTs-1	144 ± 5	27.83 ± 3.7	72.05 ± 6.7	0.98 (no fracture)
CNTs-4	280 ± 14	31.63 ± 1.8	77.91 ± 6.8	0.98 (no fracture)

σ_c,0.9_ indicates the compressive stress of the hydrogel at a strain of 0.9.

**Table 2:** The tensile properties of DN and nanocomposite DN hydrogels.

Samples	Tensile properties			

	Young’s modulus (kPa)	Yield stress (kPa)	Ultimate stress (kPa)	Ultimate strain
CNTs-0	20.8 ± 0.5	241 ± 17	681 ± 43	13.39 ± 2.08
CNTs-0.5	23.0 ± 0.3	366 ± 17	632 ± 35	11.46 ± 2.10
CNTs-1	25.6 ± 0.5	432 ± 10	630 ± 23	8.36 ± 1.77
CNTs-4	35.6 ± 0.9	533 ± 18	547 ± 28	3.45 ± 0.52

**Table 3:** Initial modulus, compressive stress, and hysteresis loss of five consecutive compressive loading-unloading cycles were given for the neat and nanocomposite.

Samples		1st	2nd	3rd	4th	5th
CNTs-0	E^a^/kPa	33 ± 2	34 ± 2	34 ± 1	37 ± 3	34 ± 2
	σ^b^/kPa	210 ± 11	199 ± 9	191 ± 1	184 ± 4	180 ± 2
	E_d_/kJ m^−3^	13.1 ± 0.8	2.3 ± 0.1	1.1 ± 0.1	1.1 ± 0.1	1.0 ± 0.1

CNTs-0.5	E^a^/kPa	77 ± 5	53 ± 7	51 ± 7	54 ± 3	57 ± 4
	σ^b^/kPa	348 ± 13	330 ± 15	317 ± 18	306 ± 15	298 ± 19
	E_d_/kJ m^−3^	23.8 ± 1.6	3.4 ± 0.2	2.9 ± 0.1	2.6 ± 0.1	2.5 ± 0.1

CNTs-1	E^a^/kPa	103 ± 7	60 ± 4	66 ± 8	65 ± 5	61 ± 7
	σ^b^/kPa	466 ± 23	451 ± 17	432 ± 14	419 ± 14	410 ± 17
	E_d_/kJ m^−3^	31.8 ± 1.8	4.5 ± 0.4	3.6 ± 0.2	3.3 ± 0.3	3.0 ± 0.2

CNTs-4	E^a^/kPa	165 ± 8	84 ± 7	85 ± 9	88 ± 7	86 ± 8
	σ^b^/kPa	478 ± 12	456 ± 16	437 ± 12	423 ± 19	415 ± 18
	E_d_/kJ m^−3^	48.4 ± 1.5	5.3 ± 0.4	3.9 ± 0.3	3.3 ± 0.2	3.2 ± 0.2

E^a^ represents the initial modulus; σ^b^ indicates the compressive stress of the hydrogel at a strain of 0.5; E_d_ represents the dissipated energy.

**Table 4:** Nomenclature and composition of nanocomposite DN gels.

Nomenclature	Composition at hydrogel preparation conditions						

	First network				Second network		
	
	C_CNT_^a^ (wt%)	C_AMPS_ (mol/L)	C_MBAA_ (mol/L)	C_KPS_ (mol/L)	C_AAm_ (mol/L)	C_MBAA_ (mol/L)	C_KPS_ (mol/L)
CNTs-0	0	1.0	0.04	0.005	2.0	0.001	0.002
CNTs-0.5	0.5	1.0	0.04	0.005	2.0	0.001	0.002
CNTs-1	1	1.0	0.04	0.005	2.0	0.001	0.002
CNTs-4	4	1.0	0.04	0.005	2.0	0.001	0.002

a C_CNT_ indicates the weight ratio of CNTs to AMPS.
